# Biomechanical characterization of human temporal muscle fascia in uniaxial tensile tests for graft purposes in duraplasty

**DOI:** 10.1038/s41598-020-80448-1

**Published:** 2021-01-22

**Authors:** Johann Zwirner, Benjamin Ondruschka, Mario Scholze, Gundula Schulze-Tanzil, Niels Hammer

**Affiliations:** 1 Department of Anatomy, University of Otago, Dunedin, New Zealand; 2 Institute of Legal Medicine, University Medical Center Hamburg-Eppendorf, Hamburg, Germany; 3 Institute of Legal Medicine, University of Leipzig, Leipzig, Germany; 4 Institute of Materials Science and Engineering, Chemnitz University of Technology, Chemnitz, Germany; 5 Institute of Anatomy and Cell Biology, Paracelsus Medical University, Salzburg and Nuremberg, Germany; 6 Department of Macroscopic and Clinical Anatomy, Medical University of Graz, Graz, Austria; 7 Department of Orthopaedic and Trauma Surgery, University of Leipzig, Leipzig, Germany; 8 Fraunhofer IWU, Dresden, Germany

**Keywords:** Ligaments, Brain, Neurosurgery

## Abstract

The human temporal muscle fascia (TMF) is used frequently as a graft material for duraplasty. Encompassing biomechanical analyses of TMF are lacking, impeding a well-grounded biomechanical comparison of the TMF to other graft materials used for duraplasty, including the dura mater itself. In this study, we investigated the biomechanical properties of 74 human TMF samples in comparison to an age-matched group of dura mater samples. The TMF showed an elastic modulus of 36 ± 19 MPa, an ultimate tensile strength of 3.6 ± 1.7 MPa, a maximum force of 16 ± 8 N, a maximum strain of 13 ± 4% and a strain at failure of 17 ± 6%. Post-mortem interval correlated weakly with elastic modulus (r = 0.255, *p* = 0.048) and the strain at failure (r =  − 0.306, *p* = 0.022) for TMF. The age of the donors did not reveal significant correlations to the TMF mechanical parameters. Compared to the dura mater, the here investigated TMF showed a significantly lower elastic modulus and ultimate tensile strength, but a larger strain at failure. The human TMF with a post-mortem interval of up to 146 h may be considered a mechanically suitable graft material for duraplasty when stored at a temperature of 4 °C.

## Introduction

Duraplasty is a common neurosurgical procedure and is required in cases where the surrounding tissue is inadequate for closure and readaption (e.g. fistula, trauma or tumor) or in cases with enlarged dural compartments such as Chiari malformation^1^. A large number of autologous^2,3^, non-autologous^4,5^, xenogeneic^5,6^ and synthetic graft materials^4^ have been used for duraplasty. None of these has so far been identified to be superior over the other materials^7^. Fascial tissues such as the temporal muscle fascia (TMF)^8^, the fascia lata^2^, the cervical fascia^9^, pericardium^10^ or pericranium^11^ are used as graft materials given their structural similarities with the collagen-rich dura mater. The TMF is the most accepted autologous graft material for duraplasty, as no additional incisions are necessary for its harvest during surgery. It is a thin and pliable tissue and can be dissected easily^8,12,13^.

In spite of this empiric experience, thorough evidence is missing on the mechanical properties of the TMF providing a suitable substitute for dura repair. Conducted mechanical experiments to date were based on small sample sizes (with a maximum of 16 tested samples) and may consequently not be representative^14–16^. Well-grounded biomechanical TMF properties may allow for mechanical comparison with dura mater, which is of interest given that the TMF transplant is exposed to deformation and flexing forces similar to the dura mater itself.

In this study, we investigated the biomechanical properties of chemically unfixed human TMF samples at a broad age range as part of the Human Head Tissue Mechanics Project. A highly standardized experimental setup for data acquisition of soft tissues has been deployed^17–19^. As a recent histological study^13^ yielded a higher content of elastic fibers in the human TMF compared with human dura mater, it is hypothesized that TMF has a higher elasticity compared to dura mater. Given that collagens are known to be subjected to age-related changes in other regions (e.g. by cross-linking or the formation of advanced glycation end-products)^20^, it is furthermore hypothesized that the mechanical parameters significantly change with age.

## Materials and methods

### Retrieval and processing of human TMF samples

**Figure 1:**
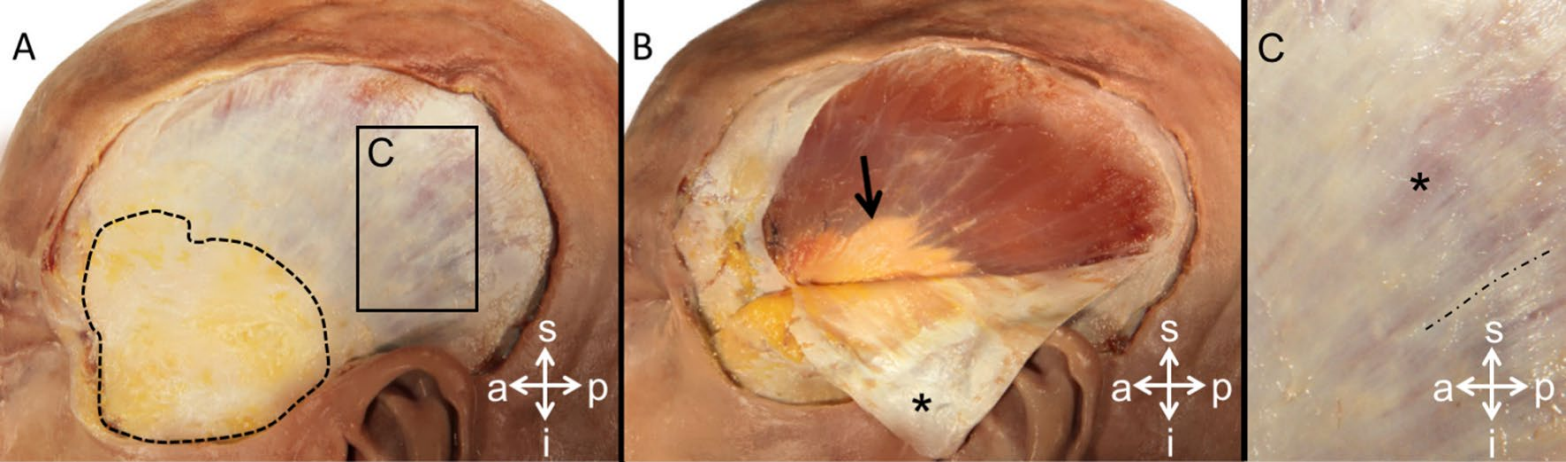
Temporal muscle fascia (TMF) of the left temporal area has been dissected in a body donor of the Department of Anatomy, University of Otago, Dunedin, New Zealand. (**A**) The superficial layer of the deep TMF becomes visible after the temporal skin and subcutaneous fat have been resected. The dotted line outlines the superficial temporal fat pad. The black rectangle represents the section of the TMF shown in (**C**). (**B**) The TMF was dissected from the underlying temporal muscle and then flipped for the inner surface of the fascia (asterisk) to become visible. Note that the inner surface of the fascia reveals a streaky pattern, indicating mechanical tissue anisotropy. The black arrow points at the temporal muscle tendon. (**C**) The anisotropic organization of the TMF can be observed in more detail. The size of the depicted image is represented by a black outlined rectangle in A. The TMF has a striated appearance with more evident areas formed by jointly-running collagen fibres over a longer distance (indicated by the dotted line) and some spot-like transparent areas (asterisk), where the temporal muscle is visible and the striated pattern is less prominent. The samples in this study were tested along the preferred collagen orientation as indicated by the dog bone shape. s, superior; i, inferior; a, anterior; p, posterior.

**Figure 2:**
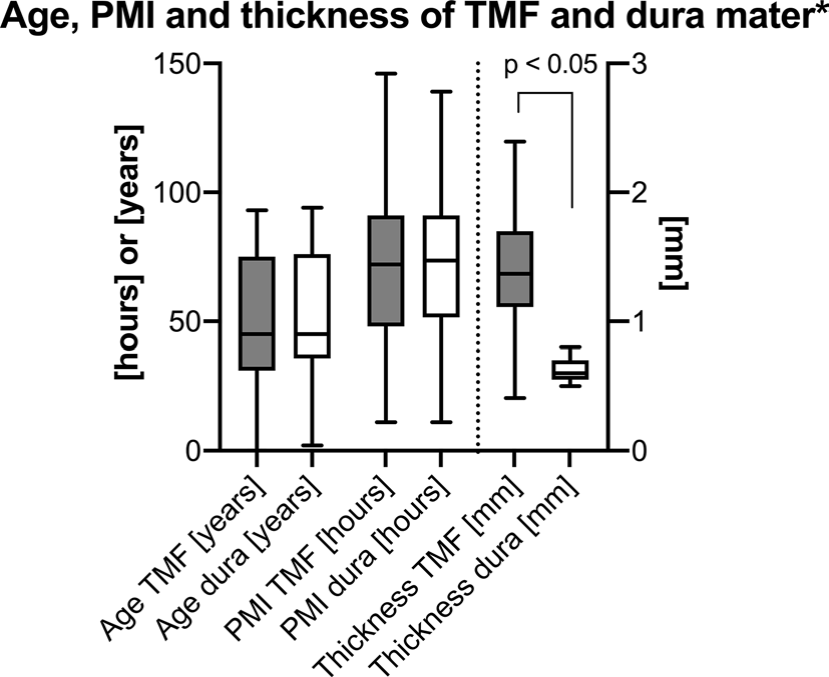
Age, post-mortem interval (PMI) and thickness of the temporal muscle fascia (TMF) samples used for biomechanical testing in this study is displayed (grey boxes). The age, PMI and thickness of recently tested dura mater samples is displayed for comparison (white boxes). The outlines of the boxes indicate the 25% and 75% percentile, the solid black line within the boxes represents the median. Whiskers indicate the minimum and maximum. *Samples tested in a different study^21^.

A total of 74 human TMF samples (23♀, 51♂; 31 left sides, 43 right sides; left and right sides from the same cadaver in 19 cases) were harvested from 55 human cadavers (18♀, 37♂) in a chemically unfixed condition at the Institute of Legal Medicine, University of Leipzig, Germany during forensic autopsy. A maximum of one sample per TMF was retrieved for the mechanical tests. Images of the TMF are displayed in Fig. 1. The samples were taken from an area of the TMF superior-posterior to the temporal fat pad and tested along the predominant collagen orientation, which was macroscopically visible as shown in Fig. 1. The samples had an averaged post-mortem interval (PMI) of 71 ± 28 h (range 11–146 h) and a mean age of 48 years (range 4 months to 93 years) (see Fig. 2). The thicknesses of the samples averaged 1.46 ± 0.41 mm (see Fig. 2). An identical age-matched (mean age 50 ± 24) number of human dura mater samples was chosen from a recent mechanical characterization of the dura mater^21^ (see Fig. 2) for subsequent comparison of the two materials. Samples were only included in this study if they were structurally unaltered, void of scars and any signs of deterioration such as greenish discoloration. Moreover, the samples had to be smoothly separable from the adjacent tissues as adhesions were treated as a sign of a previous inflammation. Head injury of the donor formed an exclusion criterion for samples to be included in this study. The thickness of the dura samples, which was determined with the Measure 2.1d software (DatInf, Tübingen, Germany) using the hardened polysiloxane mold of the tested sample’s shaft area described in detail further below, averaged 0.63 ± 0.10 mm. All methods were carried out in accordance with relevant guidelines and regulations. The protocol has been approved by the Ethics Committee of the University of Leipzig, Germany (protocol number 486/16-ek) and in line with the Saxonian Death and Funeral Act of 1994 (third section, paragraph 18 item 8). Immediately following the retrieval all samples were precooled and subsequently transferred to a − 80 °C freezer for storage.

### Water content adjustment

Samples were thawed gradually, transferring them to the −20 °C freezer for 24 h and the 4 °C fridge for an hour before further processing them at room temperature. When further processed, the samples were cut into a dog bone shaped template adapted from the ISO 527-standard (International Standard Organization, 1996). The dog bone had a clamp to clamp distance of 20 mm, a gauge length of 10 mm and a shaft width of 5 mm, as illustrated previously^22^. Small TMF subsamples of 10 cadavers (n = 40) were used to establish a tissue-specific osmotic stress protocol for the native TMF according to a previously described technique^17,21^. The small representative TMF pieces were transferred into 20 mM hydroxymethyl aminomethane-buffered polyethylene glycol (Tris-PEG; pH = 7.4; Merck KGaA, Darmstadt, Germany; molecular weight 20,000) solutions at concentrations of 2.0, 3.0, 4.0 and 5.0 wt% for 24 h. The water content of the samples was determined using lyophilization.

### Mechanical testing

Subsequent to the tapering, the TMF samples were molded with polysiloxane impression material (medium-bodied, Exahiflex; GC Corporation, Tokyo, Japan) in the area of parallel measurement length to determine their cross-sectional area as shown by Scholze et al.^19^. In brief, the liquid polysiloxane impression material was placed around the central aspect of the area of parallel measurement length and then slightly pushed against the specimen using two accurately fitting custom-made molding devices. After this, the sample was shortly placed in a moisture chamber, allowing the polysiloxane to set. Following this, the hardened polysiloxane mold was removed from the samples. The now-empty central area of the mold matching the cross-section of the shaft area of the sample was scanned at a 1200-dpi resolution (Perfection 7V750Pro; Seiko Epson Corporation, Suwa, Japan) and calculated using the Measure 2.1d software (DatInf, Tübingen, Germany). Following this, a randomly-distributed speckle pattern was created by graphite powder for the digital image correlation (DIC). Uniaxial tensile tests were conducted using a universal testing machine (Allround Table Top Z020; Zwick Roell, Ulm, Germany) at an ambient temperature of 22 °C. An Xforce P load cell of 2.5 kN with testControl II measurement electronics (all Zwick Roell) was used. A clamping jaw weighing 4.8 kg was attached to the load cell, which was defined as the new zero point. Consequently, the here used load cell with an accuracy < 1%, repeatability < 1%, reversibility < 1.5%, zero error < 1%, resolution < 0.5% (all five criteria are fulfilled from 0.4% of the maximum force) allowed for smooth force readings even below 10 N. Customized 3D-printed clamps were used for the mounting of the samples to minimize material slippage and the clamped sample was transferred to the machine using 3D-printed supporting arms, which assured a uniform clamp to clamp distance of 20 mm for each sample, as recently established by our group^19^. The former procedure assured a uniform no-load position of all samples. All samples were preconditioned with 20 load-unload cycles at a force range of 0.5–2.0 N before a stretching until failure was performed. The preconditioning aimed to assure a parallel alignment of the load-bearing extracellular matrix proteins of the TMF in the direction of load application to reduce effects of post-mortem storage, freezing and thawing on the here investigated mechanical properties. The maximum preconditioning value of 2 N was chosen as it reflected 10% of the TMF’s maximum force taken from a previous work of the group on Thiel-embalmed cadavers^23^. For the force readings, a crosshead displacement rate of 20 mm/min and a sampling rate of 100 Hz were used. The initial strain rate was 0.014 per second and has been evaluated directly by DIC in the parallel measurement length. Using a single charge-coupled camera with a resolution of 2.8 Megapixels (Q400; Limess, Krefeld, Germany), the deformation of the specimen surface was recorded perpendicular to the surface by a DIC system. For the evaluation of strain data of the mechanical tests, the ISTRA 4D software (VRS 4.4.1.354; Dantec Dynamics, Ulm, Germany) was used.

### Data processing and statistical analysis

Using MATLAB R2017b software (Mathworks, Natick, USA), the mechanical properties of the TMF samples were calculated from the DIC data and the synchronized force readings. The engineering stress was calculated nominally by the force readings and the initial cross-sectional area. The engineering strain was calculated by a virtual extensometer, which was placed in the parallel measurement length of each sample. The change of this length was related nominally to the initial length. Engineering stress–strain curves were plotted from the synchronized data and elastic modulus, ultimate tensile strength, maximum force, strain at maximum force and strain at failure were assessed by a MATLAB-routine. The elastic modulus was determined using a linear regression analysis between the zero-point and the point that equals 70% of the maximum force. The ultimate tensile strength reflects the maximum stress (maximum force divided by cross-sectional area) before the sample failed when being stretched. The maximum force refers to the peak force that the sample reached during the tensile test. The strain at maximum force reflects the sample’s strain at the point of the ultimate tensile strength in comparison to its initial length. Material failure was defined as a decrease of at least 30% of the maximum force, the corresponding strain was evaluated as strain at failure. Excel Version 16.15 (Microsoft Corporation, Redmond, USA) and GraphPad Prism version 7 (GraphPad Software, La Jolla, USA) were used for the statistical evaluation. An age-matched subgroup of our recently published mechanical characterization of the human dura mater was selected for a statistical comparison with the obtained mechanical TMF parameters^21^. The D’Agostino and Pearson normality test was used to assess Gaussian distribution of the data. Parametric data of samples were then tested using an ordinary one-way ANOVA or an unpaired t-test. The Kruskal–Wallis test or the Mann–Whitney U test were used for nonparametric data. Side comparisons between the left and right TMF were performed only for cadavers for which samples were obtained from both sides of the same cadaver using a Friedman test. This was done to exclude a potential bias due to other factors such as sex or PMI. No post hoc test has been applied, assuming the hypothesis-generating nature of this study. Age correlations of the biomechanical properties were performed for the entire age range as well as separately for the following age ranges as done previously^24^: infancy/adolescence (0–18 years), early to late middle adulthood (19–43 years) and older adulthood people (44–93 years). Pearson and Spearman correlation coefficients were reported for normally and non-normally distributed values, respectively. *P* values equal to or smaller than 0.05 were considered statistically significant. Mean values ± standard errors of the mean are reported in the text.

### Histology

Representative tissue samples of three cadavers (sex: all male, age: 68, 69 and 84 years) were fixed in 4% neutral buffered paraformaldehyde solution, dehydrated and paraffin embedded and sectioned at 7 µm according to a standard protocol. Horizontal sections were performed. These samples were then stained with hematoxylin and eosin (H&E) (Sigma-Aldrich, Munich, Germany). A DM1000 LED light microscope (Leica, Wetzlar, Germany) was used to take photos.

## Results

### Biomechanical properties of human TMF

**Figure 3:**
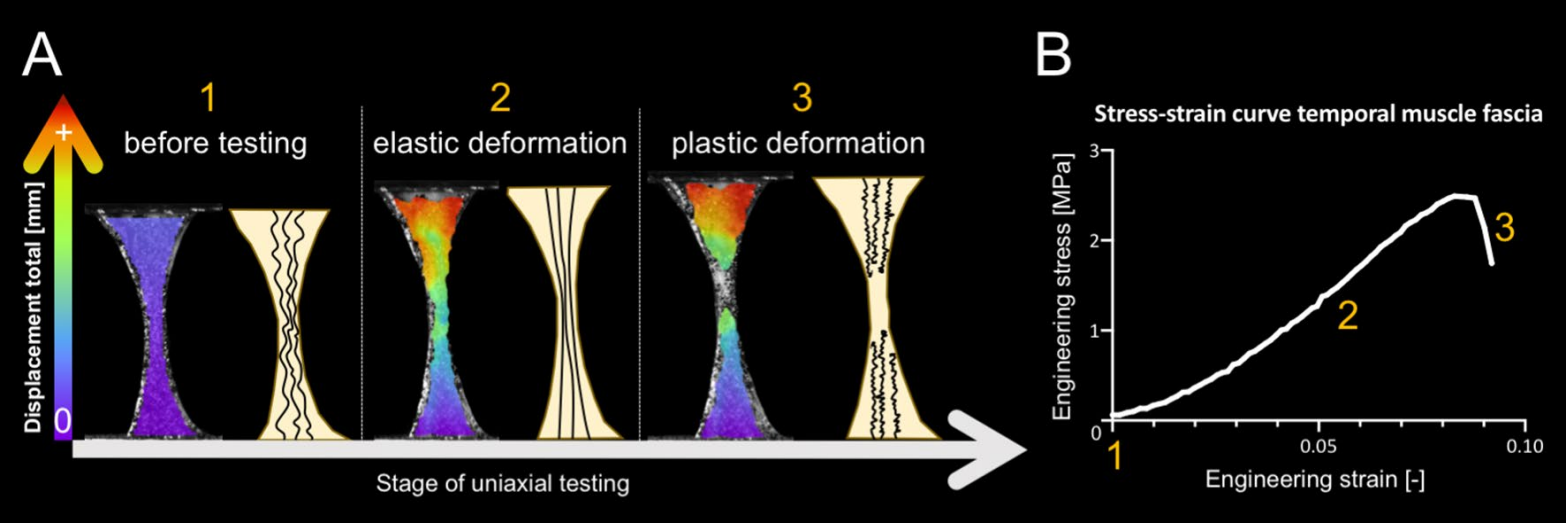
Images of a representative temporalis muscle fascia (TMF) sample at different stages of uniaxial tensile testing with a schematic depiction of the related collagen behavior and the related stress–strain curve are displayed. (**A**) The TMF sample is shown before the load is applied (1). The collagens within the sample are in a crimping state. A uniform diminution occurs in the tapered area of the tested sample once a load is applied. When the sample is elastically deformed the collagens are stressed (2). When the TMF is stressed beyond its yield point, it will deform plastically, schematically represented by collagen rupture (3). (**B**) The related stress–strain curve of the sample shown in A is depicted. The orange numbers correspond to the stages of testing, equally represented by orange numbers in **A**.

**Figure 4:**
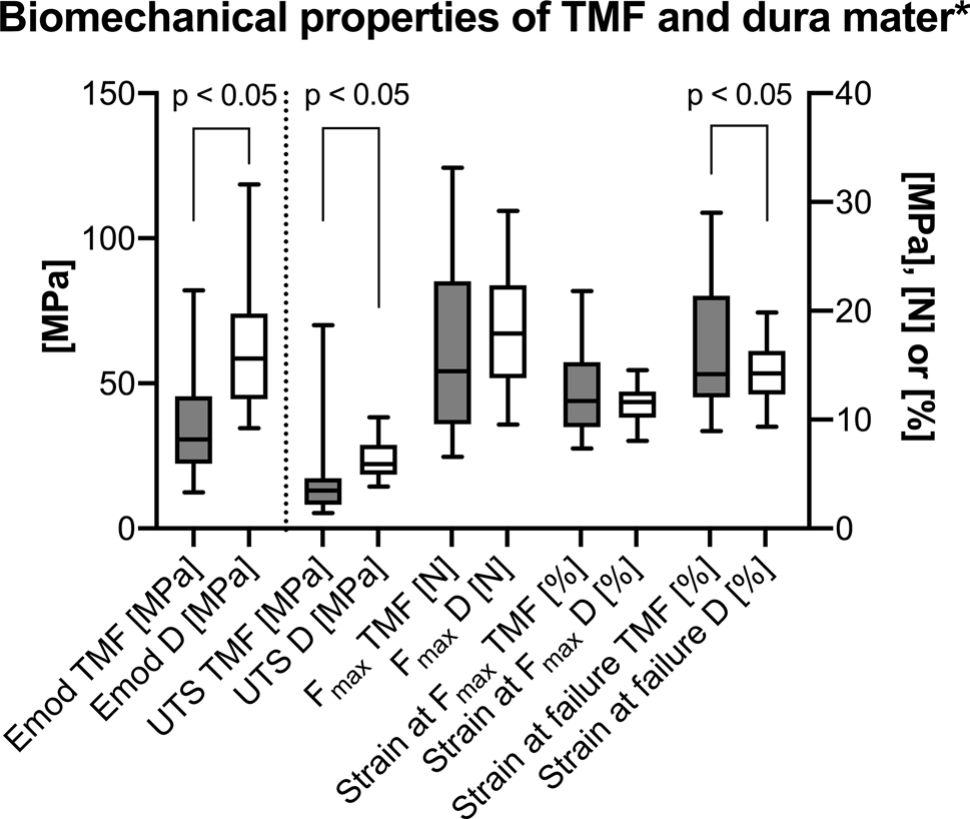
The biomechanical parameters of native temporal muscle fascia (TMF) and an age-matched group of dura mater (D) samples are displayed. The outlines of the boxes indicate the 25% and 75% percentile, the solid black line within the boxes represents the median. Whiskers indicate the minimum and maximum. The dotted line separates left and right y-axis. Emod, elastic modulus; F_max_, maximum force; S_failure_, strain at failure; SF_max_, strain at maximum force; UTS, ultimate tensile strength; * values taken from Zwirner et al. 2019^21^.

TMF had an elastic modulus of 36 ± 19 MPa, an ultimate tensile strength of 3.6 ± 1.7 MPa, a maximum force of 16 ± 8 N, a maximum strain of 13 ± 4% and a strain at failure of 17 ± 6%. See Fig. 3 for images of the tensile testing and a representative stress–strain curve and Fig. 4 for a graphical depiction of the mechanical properties of the human TMF. The age-matched dura samples had an elastic modulus of 63 ± 21 MPa, an ultimate tensile strength of 6.4 ± 1.8 MPa, a maximum force of 18 ± 6 N, a maximum strain of 11 ± 2% and a strain at failure of 14 ± 3%. The mechanical dura mater properties are depicted in Fig. 4 for comparative reasons. The elastic modulus (*p* = 0.003) and the ultimate tensile strength (*p* = 0.017) were significantly lower for the TMF, whereas the strain at failure was significantly higher (*p* = 0.003) compared to the dura mater. There was no difference on a statistically significant level regarding the maximum force (*p* = 0.331) and the strain at maximum force (*p* = 0.065) between the TMF and the dura mater. However, the TMF samples in this study were significantly thicker compared to the dura samples (*p* < 0.001), but did not significantly differ with regards to their PMI (*p* = 0.699).

### Elastic modulus and failure strain of human TMF depend on the PMI, but are independent of age

**Figure 5:**
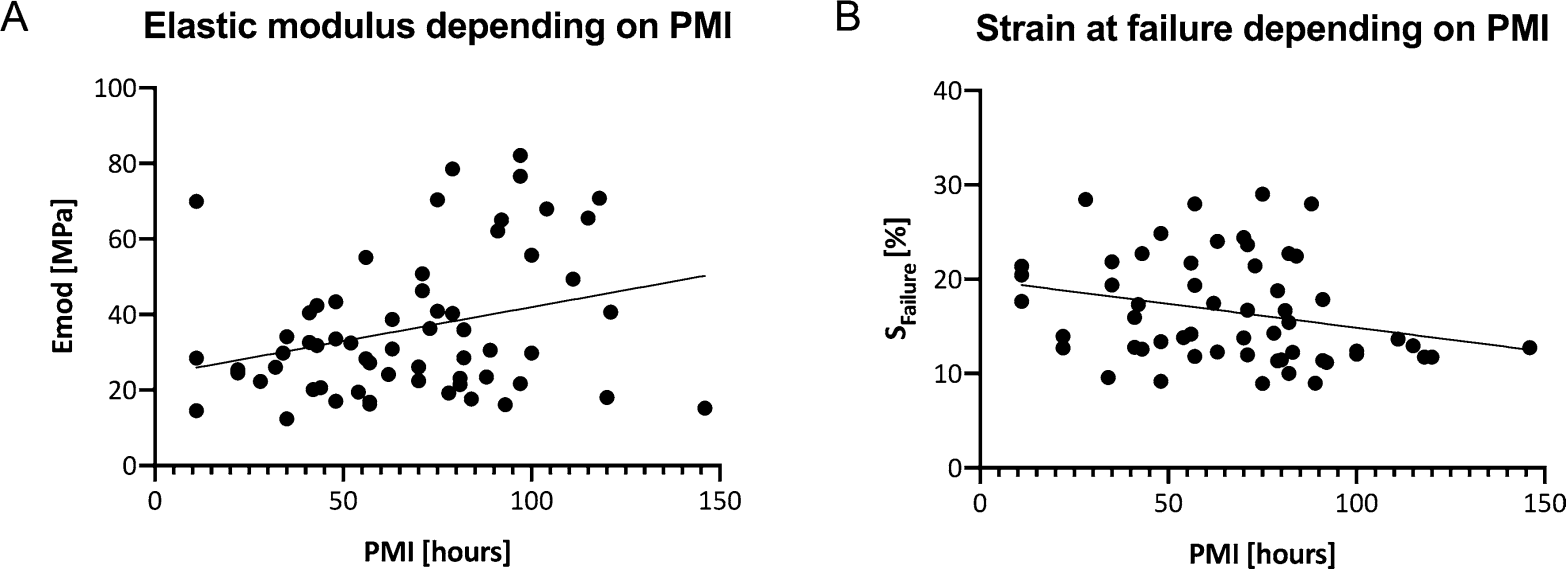
Post-mortem interval (PMI)-dependent biomechanical parameters of the temporal muscle fascia (TMF). (**A**) The elastic modulus of the TMF significantly increases with longer PMIs. (**B**) The strain at failure of the TMF significantly decreases with longer PMIs. Emod, elastic modulus; S_failure_, strain at failure.

**Figure 6:**
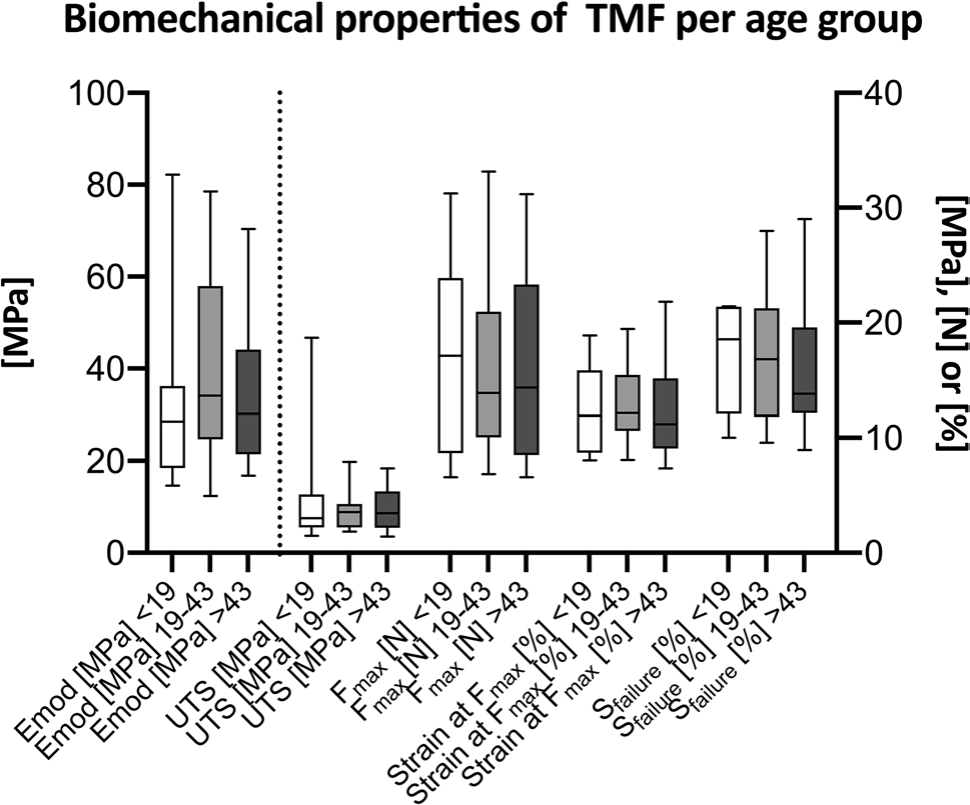
The biomechanical properties are depicted separately for the following three age groups: infancy/adolescence (0–18 years), early to late middle adulthood (19–43 years) and older adulthood people (44–93 years). The dotted line separates the left and right y-axis. Emod, elastic modulus; F_max_, maximum force; S_failure_, strain at failure; SF_max_, strain at maximum force; UTS, ultimate tensile strength.

The elastic modulus of the TMF significantly correlated weakly positive with the PMI of the tested samples (r = 0.255, *p* = 0.048) (see Fig. 5A). Moreover, a weak but significant decrease was shown for the strain at failure and PMI (r =  − 0.306, *p* = 0.022)(see Fig. 5B). None of the other mechanical parameters revealed significant correlations with PMI. Age did not correlate on a statistically significant level with the biomechanical parameters of the TMF (*p* ≥ 0.24). The former was also true when the biomechanical parameters were correlated separately for the age groups of < 19 years, between 19 and 43 years and > 43 years (see Fig. 6). Moreover, all aforementioned biomechanical parameters were statistically non-different between the left and the right TMF (*p* > 0.999 for all parameters).

### Male TMFs were significantly less elastic when compared to females

**Figure 7:**
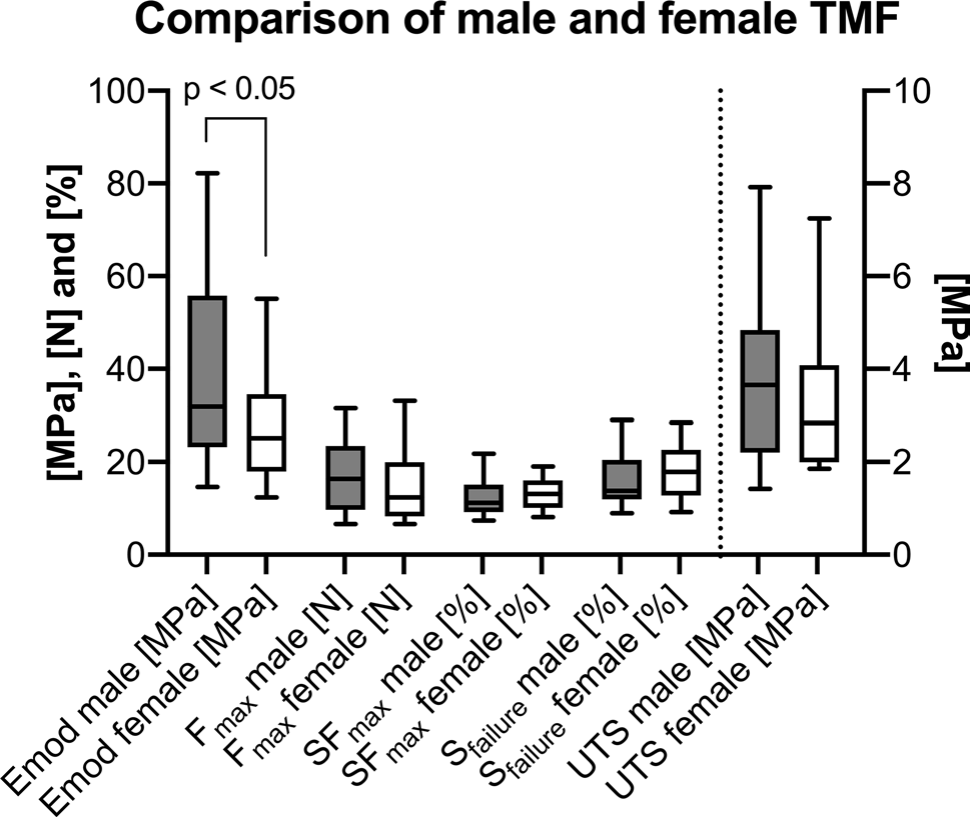
The biomechanical properties of the temporal muscle fascia (TMF) are depicted separately for males and females. The elastic modulus of TMFs harvested from male cadavers was significantly larger compared to female ones. The outlines of the boxes indicate the 25% and 75% percentile, the solid black line within the boxes represents the median. Whiskers indicate the minimum and maximum. The dotted line separates left and right y-axis. Emod, elastic modulus; F_max_, maximum force; SF_max_, strain at maximum force; S_failure_, strain at failure; UTS, ultimate tensile strength.

The averaged elastic modulus of male TMFs (40 ± 20 MPa) was significantly higher compared to the female ones (28 ± 11 MPa; *p* = 0.003). There was no significant difference between sexes regarding the ultimate tensile strength (male: 3.7 ± 1.7 MPa, female: 3.2 ± 1.6 MPa; *p* = 0.897), maximum force (male: 17 ± 8 N, female: 15 ± 8 N; *p* = 0.535), strain at maximum force (male: 13 ± 4%, female: 13 ± 6%; *p* = 0.848) nor strain at failure (male: 16 ± 5%, female: 18 ± 6%; *p* = 0.557). Moreover, there was no statistically significant difference regarding male (73 ± 30 h) and female (67 ± 25 h) PMI (*p* = 0.076). The biomechanical TMF parameters are separately displayed for males and females in Fig. 7.

### H&E staining of TMF

**Figure 8:**
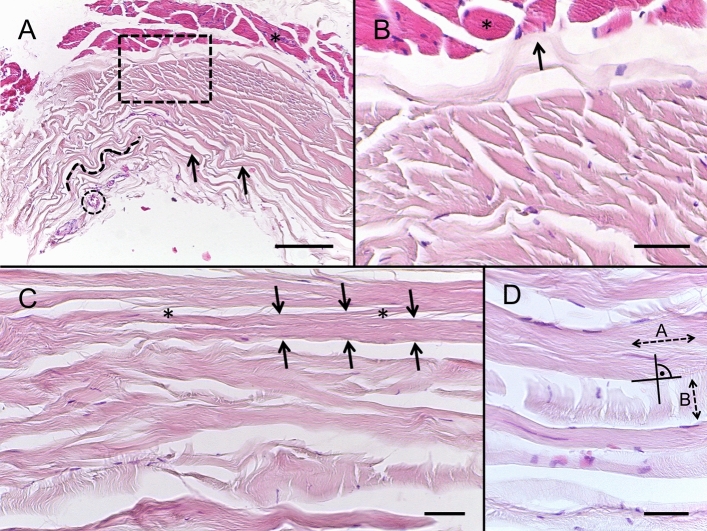
Haematoxylin & eosin images of the temporal muscle fascia (TMF). (**A**) A horizontal section of the TMF is depicted covering the temporal muscle (asterisk). TMF consists of collagen bundles (black arrows) organized in several layers. In several parts, a crimping pattern of the collagen bundles was observed (dotted line). A frontal branch of a superficial temporal vessel is shown (dotted circle). Magnification: 100 × , scale bar: 100 μm. (**B**) The image corresponds to the box shown in A and displays the transition between the TMF and the temporal muscle (asterisk). The TMF is attached to the temporal muscle with its innermost collagen layers. This discontinuous attachment had to be cut sharply when the TMF was removed from the muscle for biomechanical tests conducted in this study. Magnification: 200 × , scale bar: 50 μm. (**C**) At a higher magnification it could be observed that the collagen bundles (black arrows indicate a collagen bundle) were sparsely interconnected (asterisks) among each other over longer distances. Magnification: 200 × , scale bar: 50 μm. (**D**) The predominant collagen bundles (dotted arrow marked with **A**) were connected by a perpendicular running thin collagenous layer (reflected by the dotted arrow marked with **B**). Magnification: 400 × , scale bar: 50 μm.

The H&E staining confirmed that the TMF is composed of collagen bundles organized in several layers. These layers seemed poorly interconnected. A crimping pattern of the collagen bundles was seen. The innermost collagen layer of the TMF was discontinuously attached to the temporal muscle. A thin layer of collagens, orientated in a perpendicular fashion to the predominantly running collagen bundles, interconnected these collagen bundles (Fig. 8).

## Discussion

The TMF combines several advantageous features as a duraplasty graft, ideally, including reduced immunogenicity, nontoxicity, pliability, potentially a rapid integration into the transplant host site and its anatomical location making this an easy-to-harvest and inexpensive material^7^. The TMF has been reported to provide a material of reasonable tissue strength^7^, which can be confirmed based on our large-scale mechanical analysis presented here. The collagen backbone of TMF not only crucially defines these mechanical properties, but also provides a suitable environment for the migration and differentiation of cells after the TMF has been transplanted^25^ with subsequent extracellular matrix remodelling. Especially in supratentorial dura mater lesions, the TMF seems to be a beneficial graft option as it can be harvested via the primary exposure and thus does not require an additional surgical approach for graft removal^8^.

The averaged elastic modulus of the here tested TMF is significantly lower compared to the dura mater samples from a similar cohort, tested in an identical testing setup^21^. Thus, we accept our first hypothesis and state that human TMF is a more elastic material compared to human cranial dura mater. This is in accordance to higher contents of elastic fibers noted in the TMF compared to dura mater stated by Morales-Avalos et al.^13^. The elastic behavior of any given graft for dura mater repair is of importance as the material will be mechanically stressed at the host site by means of cerebrospinal fluid pulsations, which has been stated for expansile duraplasties^9^. In this regard, a more elastic graft like the TMF would be beneficial as it allows for larger graft expansions during the cerebrospinal fluid pulsations and for more laminar cerebrospinal fluid flow similar to the chamber flow mechanism of the aorta, potentially serving as a reserve space that protects the brain from damage, resulting from increased intracranial pressure. The intracranial pressure was shown to be related to dura mater material properties in finite element simulations^26^. Such important and experimentally-substantiated considerations need to be considered strongly for any transplant material such as the here-investigated TMF. However, with regards to the multiaxial nature of cerebrospinal fluid pulsations at dynamic strain rates in vivo it has to be noted that the here stated uniaxial quasi-static testing setup cannot fully describe the behavior of the TMF graft in vivo. The elasticity of the TMF might be beneficial when being used for bridge-like duraplasty, which may to some extent mimic a physiological biomechanical behavior of the brain-skull composite^27^. Similar to the response during cerebrospinal fluid pulsations, the collagens of the TMF graft can store and dissipate energy^28^ during head movements and postural changes. Mechanical resistance of the TMF to the loads of the head and brain are exerted to form another relevant aspect, and this should be compared to other duraplasty grafts including the dura mater. Regarding this, the ultimate tensile stress before the TMF mechanically failed has been significantly lower compared to the tensile strength obtained for native dura mater^21^. This should be respected in a clinical context when the TMF is used as a dura graft. Here, the function of the tissue reaches beyond an impermeable membrane to also serve as an equally load-resistant graft. Future biomechanical studies should explore whether a folded and thus multi-layered TMF graft would be better suited to withstand higher stresses that equal or exceed the ones reported for human dura mater. However, the implication of folding of the TMF in duraplasty on the other abovementioned biomechanical parameters then also has to be explored in depth. The thickness of the TMF samples observed in this study of 1.46 ± 0.41 mm was larger compared with the site-dependent dura thicknesses of 0.36 ± 0.16 mm (petrous apex)^29^, 0.68 ± 0.20 mm (temporal dura presented in this study as a subgroup of recent investigations^21^), 0.91 ± 1.14 (unspecified area)^13^ or 1.11 ± 0.24 mm (craniocervical junction)^30^. However, it has to be noted that the evaluation of dura mater thickness by other research groups has partly been performed on embalmed tissue^13^, likewise introducing a significant bias. The varying collagen architecture between the TMF and the dura mater forms most likely the reason for the observed mechanical difference of the two tissues. The structural analyses performed in this studys displays that the TMF is composed of multiple collagen layers organized in aligned fiber bundles. However, these bundles run in an anisotropic fashion, similar to the temporal muscle fibers, and are sparsely interconnected. The former indicates that the TMF is repetitively loaded in a similar fashion during masticatory motions by the underlying temporal muscle. The collagens of the five-layered human dura mater^31^ are organized in a more complex way, which might be simplified as a mesh-like structure with several highly-aligned strengthening lines^32^. The presumably stronger three-dimensional interconnection of the collagen backbone seen in the dura mater^21^ might be the reason for the observed higher mechanical strength of dura compared to TMF^31,32^. The tensile loads in this study were applied according to the predominant axis of the collagens. The strain behavior of a tissue, which has a certain number of collagens running perpendicular to each other by being closely linked (such as the dura mater)^21^ will likely be reduced. Thus, it can be explained why the TMF showed a significantly increased failure strain compared with the dura mater^21^.

In this study, we observed that with an increasing PMI the TMF becomes less elastic and loses straining potential. This might be the consequence of increased collagen cross-linking with an increased PMI (time between death of the cadaver and harvesting of the TMF)^33^, but not the effect of tissue water loss given the tissues used here were osmotically adapted. After death, biomechanical changes occur in all body tissues as a consequence of the oxygen undersupply^34^. For example, the temporal muscle, which is in proximity to the TMF, is likely to show increased levels of lactic acid after death, as a result of the anaerobic glycolysis^34^. As a consequence of high lactate levels (decreasing pH levels), the occurring biochemical changes after death might biomechanically influence the flexible sites (sequences lacking the amino acids proline and hydroxyproline) of the collagen backbone^28^, resulting in stiffer and less extensible collagen fibrils. However, these potential post-mortem changes did not affect the overall strength (ultimate tensile strength and maximum force) of the TMFs of this study, which indicates that the integrity of the collagen fibrils in general is not significantly damaged within a 146-h time tested here. The post-mortem mechanical alterations of TMF presented here are unlikely to affect the duraplasty graft even over an extensive time frame of several days at temperatures not exceeding 4 °C, which may have implications for the use of cadaveric TMF for transplantation purposes.

This given study investigated the biomechanical properties of the human TMF in relation to sex and age. The comparison of the load-deformation properties between males and females showed no significant difference except for elastic modulus, being higher in males. Generally, masticatory behavior between males and females was shown to be different before^35^. Males presented higher biting forces^36^ and had larger facial dimensions including thicker temporal muscles from an anatomical perspective^35,37^. Thus, the TMF of males might be strained differently compared to females, resulting in different elastic moduli. The collagen synthesis by fibroblasts is influenced by male and female sex hormones^38,39^. Moreover, the investigation of wound healing showed different effects of male and female sex hormones^40^. Additionally, no age-dependency of biomechanical parameters was observed in the here investigated age range. Consequently, we reject our second hypothesis. The constant need for food intake, and thus mechanical stimulation of the TMF during the entire life span might be a possible explanation that mechanical parameters are independent of age.

Biomechanical properties of the human TMF have barely been investigated before^14–16^ with a maximum of 16 tested TMF samples very recently^15^. Puksec et al. tested TMF samples using a uniaxial setup and a displacement velocity of 0.05 mm/s^15^. As revealed in this given study Puksec et al. stated a more elastic behavior of the TMF as compared to the dura mater samples’ elastic limit of 6% strain, however, without reporting an elastic modulus^15^. The ultimate tensile strength of TMF in their study of 3.9 MPa could be substantiated in our study, investigating about fourfold their sample size and a broader age range under standardized conditions. Curtis et al.^14^ testing at a constant load rate of 2 N/min reported a much larger modulus of 148 MPa compared to the here obtained elastic modulus, which shows no overlap with our data or the values presented elsewhere. However, only four samples of one adult male embalmed human cadaver were tested in their study^14^. Their vastly limited sample size and the embalming chemicals will have likely dehydrated and denaturized the tissues, which results in higher elastic moduli^41^. The thicknesses of human TMF, dura mater and scalp samples were compared between Thiel-embalmed tissues and unembalmed tissues of the same origin before^23^. All embalmed tissues were consistently thinner compared to the unembalmed ones^23^. However, this relationship was only statistically significant for scalp samples^23^. As the former study only involved six cadavers per tested group, a potential dehydration of embalmed tissues compared to unembalmed ones should be explored in depth using a larger sample size in the future. The group of Trindade et al.^16^ investigated 16 samples from eight cadavers at a deformation rate of 5 mm/min and reported a secant modulus between 0.14 (20 to 50 years) and 1 MPa (51 to 70 years), which they defined as a ratio of Cauchy stress to a corresponding 10%-deformation^16^. The tensile strength in their study was between 1.14 MPa (20 to 50 years) and 7.58 MPa (51 to 70 years), which does not concur with our results in a much larger sample size and a standardized setup. The considerable scatter in tensile strength values reported by Trindade et al.^16^ might be explained, potentially, by a non-uniform testing orientation of the samples. The here given work as well as other previous mechanical characterizations of the human TMF^14,15^ orientated the samples longitudinally to the predominant collagen orientation, and may in consequence lead to more uniform ultimate tensile strength values. As a consequence of the lacking and inconsistent biomechanical properties in literature, the TMF has so far been neglected in computational and physical models of the temporomandibular joint or the entire human head^42–49^. The biomechanical description of the human TMF in this given study contributes to the implementation of the TMF into future human head models.

### Limitations

A quasi-static uniaxial tensile testing setup was chosen for the TMF tensile tests. Under native conditions, the loading is likely to be of a more dynamic and multiaxial nature. Minor but macroscopically invisible disruptions of the extracellular matrix might have been caused once the TMF was carefully removed from the underlying temporal muscle, with potential effects on the biomechanical properties of the tested samples. The deep-freezing of fresh tissues subsequent to the retrieval as performed in this study for storage purposes may have affected the here-obtained biomechanical parameters. However, previous studies that investigated the effect of deep-freezing at −80 °C on the biomechanical parameters of collagen-rich tissues revealed inconclusive results^50–52^. Further to this, a protocol including step-wise thawing was used aiming to minimize the buildup of ice needles with potential deterioration of the extracellular matrix backbone in the tissues. Future studies may add to these findings, examining tissues right after death without deep-freezing in comparison, thereby exploring the effects of deep-freezing on the here investigated biomechanical properties of the human TMF. Preconditioning of biological tissues for the aforementioned purposes is commonly done in tensile testing protocols as performed here^52–54^. However, the detailed effect of preconditioning on the biomechanical properties of biological tissues yet remains to be investigated in detail.

## Conclusion

Human TMF is significantly more elastic and shows a higher straining potential compared to the human dura mater, but is less resistant in a uniaxial quasi-static loading setup. No signs of TMF graft degeneration were observed for a 146-h post-mortem time frame from a mechanical perspective when the tissues are stored at a maximum of 4 °C. Age did not influence the biomechanical properties in an age range of 4 months to 93 years relevantly, indicating TMF to be a suitability graft material irrespective of donor age even in post-mortem tissue donation programs.
